# Low level of BRCA2 in peripheral blood lymphocytes is associated with an increased risk for head and neck squamous cell carcinoma (HNSCC) in a population of North-East India: a case-control study

**DOI:** 10.3332/ecancer.2023.1503

**Published:** 2023-02-02

**Authors:** Sambuddha Das, Aditi Bhowmik, Abhinandan Bhattacharjee, Biswadeep Choudhury, Momota Naiding, Bishal Dhar, Agniv Kr Laskar, Jayeeta Pandit, Sankar Kumar Ghosh, Yashmin Choudhury

**Affiliations:** 1 Department of Biotechnology, Assam University, Silchar 788011, India; 2 Department of ENT, Silchar Medical College and Hospital, Silchar 788014, India; 3 Department of Biochemistry, Silchar Medical College and Hospital, Silchar 788014, India; 4 Department of Pathology, Silchar Medical College and Hospital, Silchar 788014, India

**Keywords:** APE1, BRCA2, CART analysis, HNSCC risk, North-East India, XPD

## Abstract

**Background:**

We investigated the role of DNA repair proteins breast cancer susceptibility 2 (BRCA2), xeroderma pigmentosum group D (XPD) and apurinic/apyrimidinic endodeoxyribonuclease 1 (APE1) in determining the risk for head and neck squamous cell cancer (HNSCC) in a case-control study from North-East India.

**Methods:**

Expression of *BRCA2*, *XPD* and *APE1* genes in the matched tumour, normal adjacent tissue (NAT) and blood of 12 HNSCC patients and blood of 8 age- and gender-matched controls was determined by quantitative real-time PCR. Results were validated by expression analysis of the corresponding proteins in the peripheral blood lymphocytes (PBLs) of 228 subjects (106 patients and 122 controls) by a slot-blot immunoassay.

**Findings:**

Expression of the *BRCA2* and *XPD *genes in tumour tissue of HNSCC patients declined progressively as the cancer stage advanced, was reverse that of the NAT, but was mirrored by the expression in the blood. BRCA2 and XPD proteins were significantly (*p* < 0.0001) downregulated in the PBL of HNSCC patients to 71% and 77% the levels in controls, showing significant negative correlation with HNSCC stage (Spearman correlation coefficient (*r*_s_) of −0.9060, *p* < 0.0001 for BRCA2; *r*_s_ of −0.8008, *p* < 0.01 for XPD). On the contrary, APE1 was significantly upregulated in PBL of HNSCC patients to 1.47 fold the level in controls, showing significant positive correlation with HNSCC stage (*r*_s_ of 0.7023, *p* < 0.01). Classification and regression tree analyses predicted low levels of BRCA2 protein in PBL as the single most important risk factor for HNSCC, independent of gender. Smokers above 36 years of age with low level of BRCA2 appeared to exhibit a 1.78-fold increased risk for HNSCC (with a 1.78-fold increased risk for HNSCC (OR = 1.78, 95% confidence interval (CI) = 0.33–9.52) though this risk was not significant statistically. Similarly, low levels of BRCA2 appeared to indicate a moderate, but non-significant risk for HNSCC in non-smokers aged between 36 and 56 years (OR = 1.15, 95% CI = 0.21–6.37).

**Conclusions:**

Low level of BRCA2 protein in the peripheral blood indicates increased risk for HNSCC.

## Introduction

Head and neck squamous cell carcinoma (HNSCC) comprises cancers derived from the mucosal epithelium of the oral cavity, pharynx and larynx. The aetiological factors generally associated with HNSCC are tobacco use, alcohol use and infection with high risk types of human papillomavirus (HPV) [1]. Tobacco use is also a risk factor for cancer recurrence, poorer treatment response and treatment related toxicity [2]. Cigarette smoke contains polycyclic aromatic hydrocarbons and carcinogenic compounds such as 4-methylnitrosoamino-1-(3-pyridyl)-1-butanone (NNK), N-nitrosonornicotine (NNN), radon, formaldehyde, acrolein, acetaldehyde, 1,3 butadiene and benzene, several of which form adducts on DNA [3, 4]. Besides smoking, various forms of smokeless tobacco such as *khaini*, *zarda*, *gutkha* and *betel quid* (BQ) are commonly used in some countries of South-east Asia such as India, Bangladesh and Nepal [5]. These products also contain DNA-adduct forming nitrosamines such as NNN and NNK [6].

DNA damage caused by tobacco-derived carcinogens is repaired by various DNA repair pathways which maintain genomic integrity and stability. The helix-distorting bulky adducts formed by benzo(a)pyrene, NNN and NNK are repaired by nucleotide excision repair (NER), oxidative damage induced by reactive oxygen species is repaired by base excision repair (BER) [7] and double-strand breaks (DSB) resulting from use of smokeless tobacco [6] or exposure to acetaldehyde in tobacco smoke [8] are repaired by homologous recombination (HR).

The North-Eastern Region of India has the highest incidence of cancer in the country. Notably, 50% of all cancers in men and 28% of all cancers in women in the region are tobacco-related cancers owing to the rampant use of both smoked and smokeless tobacco in the region [9]. A previous study has reported that tobacco and alcohol consumption in the region may be associated with high-risk HPV infection, which is an important risk factor particularly for oral and oropharyngeal cancers [10].

We have previously reported that xeroderma pigmentosum Group D (*XPD*), apurinic/apyrimidinic endodeoxyribonuclease 1 (*APE1*), mut Y DNA glycosylase [11] and murine double minute 2 homolog [12] polymorphisms increase HNSCC risk in the North-East Indian population, and expression of the breast cancer susceptibility 1 protein is downregulated in peripheral blood lymphocytes (PBLs) of HNSCC patients of the region, with potential for acting as an independent, blood-based biomarker of HNSCC [13]. On the basis of our earlier findings, we hypothesised that another crucial effector of the HR pathway, breast cancer susceptibility 2 (BRCA2), along with the XPD and APE1 protein of the NER and BER pathways, respectively, may play a vital role in determining the risk for HNSCC in association with tobacco use, in the population of the region. We also proposed that if circulating blood cells can mirror the expression level of DNA repair genes in tumour tissue of HNSCC patients, they may be utilised for non-invasively evaluating the risk of an individual for HNSCC. We tested our hypothesis by evaluating the expressions of the BRCA2, XPD and APE1 genes and proteins in tissue biopsies and blood samples of study subjects drawn from the high-risk North-East Indian population, and their correlation with various aetiological factors and clinicopathological features of HNSCC.

## Material and methods

### Ethics approval

All the procedures performed in this study were in accordance with the ethical standards of the institutional and/or national research committee and with the World Medical Association Declaration of Helsinki (version 2002) and its later amendments or comparable ethical standards. Approval for the study was obtained from the Institutional Review Board of Silchar Medical College and Hospital (SMCH), Silchar (No. SMC/11/7932-41 dated 19/7/2011) and Institutional Ethics Committee (IEC) of Assam University, Silchar (No. IEC/AUS/2013-009 dated 20/3/2013). Samples were collected and processed between 2013 and 2015.

### Subjects

Our study comprised a total of 228 study subjects. Among these, 106 patients (68 males and 38 females) with histologically proven premalignant or malignant HNSCC hailing from different parts of North-East India admitted at the Department of Ear, Nose, Throat, SMCH, Silchar for treatment. The cancers were staged and graded at the Department of Pathology, SMCH, following the tumour-node-metastasis cancer staging system prescribed by the The American Joint Committee on Cancer and the International Union for Cancer Control for cancers diagnosed on or after 1 January 2010 [14]. A total of 122 volunteers (75 males and 47 females) unrelated to the patients, without a prior history or any family history of cancer and matching the patients with respect to gender and age-group were included as control subjects in this study. The HNSCC patients and controls were grouped on the basis of age into the following groups: 11–20, 21–30, 31–40, 41–50, 51–60, 61–70, 71–80, 81–90 and were matched accordingly. Study subjects were interviewed on the oral habits of smoking, chewing tobacco (CT) use and betel-nut (BN) chewing through a standard questionnaire, and were classified into the following categories based on the dose of use: (a) moderate smokers who smoked <20 cigarettes/*bidis* per day, (b) heavy smokers who smoked ≥20 cigarettes/*bidis* per day, (c) moderate CT + BN chewers who chewed <10 units of *guthka*/*khaini*/BQ per day and (d) heavy CT + BN chewers who chewed ≥10 units of *guthka*/*khaini*/BQ per day.

Venous blood (5 mL) was collected from all controls and HNSCC patients with informed consent. Patients did not receive either chemotherapy or radiotherapy before the blood was drawn. Matched tumour tissue and normal adjacent tissue (NAT) were also obtained from 12 of these patients (8 males and 4 females) with informed consent. Blood as well as tissue samples were taken from patients prior to chemotherapy or radiotherapy. Informed consent was obtained from the patients as a standard consent form in the local language.

### Sample collection

Venous blood (5 mL) was drawn from HNSCC patients and controls by medical practitioners of SMCH, with informed consent, and collected in sterilised ethylenediaminetetraacetic acid (EDTA) vials. Immediately after collection, 300 μL of blood was mixed in equal volume of TRIzol and stored refrigerated at −86°C to be used for RNA isolation. The remaining blood samples were processed for separation of PBL, and preparation of PBL lysate for western blotting/slot blotting on the day of collection itself. Tumour tissue and NAT from HNSCC patients were retrieved by a surgeon at SMCH, kept in sterile vials containing TRIzol and stored refrigerated at −86°C, to be used for RNA isolation.

### Chemicals and reagents used

All chemicals and reagents used in this study were either of analytical grade or molecular biology grade and were used without further purification. The chemicals and reagents used were as follows:

Ethidium bromide, glycine, Histopaque 1077, sodium azide (NaN_3_) and protein-A Agarose beads (P1406), proteinase K, secondary antibody alkaline phosphatase-labelled goat anti-rabbit IgG (A 9919, polyclonal antibody) and primers for real-time PCR were procured from Sigma-Aldrich, USA. Taq polymerase, 1× PCR buffer were purchased from Promega (Promega, USA), deoxynucleoside triphosphate (dNTP) mix was procured from Fermentas (Fermentas, USA). Ammonium persulfate, bovine serum albumin, Coomassie Brilliant Blue G250, 2-mercaptoethanol, acetic acid glacial, magnesium chloride (MgCl_2_), phenylmethanesulfonyl fluoride were obtained from Sisco Research Laboratories Pvt. Ltd., India. Acrylamide, agarose, 5-bromo-4-chloro-3-indolyl phosphate/nitro blue tetrazolium chloride (BCIP/NBT), di-sodium hydrogen phosphate, EDTA, methanol, N,N-methylene bisacrylamide, N,N,N N-tetramethylenediamine, ortho-phosphoric acid, potassium biphosphate, potassium chloride, sodium acetate, sodium chloride, phenol, chloroform, isoamyl alcohol, sodium dodecyl sulphate (SDS), sodium hydroxide, Tris base (hydroxymethyl) aminomethane, Triton X-100, Ponceau S sodium salt and Tween-20 were procured from HiMedia Laboratories Pvt. Ltd., India. Ethanol was procured from Bengal Chemicals and Pharmaceuticals Ltd., Kolkata, India. Protease inhibitor cocktail (M 222) was obtained from AMRESCO LLC, USA. Skimmed milk powder (fat free) was procured from Amul, India. Polyvinyl difluoride (PVDF) membrane was obtained from Santa Cruz Biotechnology, Inc., USA. TRIzol® Reagent, RevertAid First Strand cDNA Synthesis Kit was obtained from Thermo Scientific^TM^, USA. Power SYBR Green PCR Master Mix was procured from Applied Biosystems, USA. Primary antibodies anti-BRCA2 (ab27976), anti-XPD (ab167418), anti-APE1 (ab137708) and Prism ultra protein ladder 10–245 kDa (ab116028) were obtained from Abcam, UK. Primary antibody anti-β-actin (N-21, polyclonal IgG) was obtained from Santa Cruz Biotechnology, Inc., USA. All primary antibodies procured were raised in rabbit. All buffers and reagents were prepared in double-distilled water.

### Isolation of RNA and quantitative real-time PCR

Total RNA was isolated from the blood, tumour tissue and NAT of 12 HNSCC patients and the blood only of 8 controls using TRIzol reagent, (Thermo Scientific, USA). This was followed by c-DNA synthesis using oligo-dT primers (Thermo Scientific, USA) and reverse transcriptase kit (RevertAid First Strand cDNA Synthesis Kit, Thermo Scientific, USA). The purity and concentration of the c-DNA was determined by spectrophotometric readings using SmartSpec Plus Spectrophotometer (Bio-Rad, USA) based on assessment of the OD_260/280_ ratio of the cDNA sample. Accordingly, only samples with an OD_260/280_ ratio between 1.8 and 2.0 were included for further study. The expressions of *BRCA2*,* XPD* and *APE1 g*enes were evaluated by real-time PCR using SYBR Green PCR kit (Applied Biosystems, USA) and gene specific primers (Sigma-Aldrich, USA) (Supplementary Table 1) on StepOne Real-Time PCR system (Applied Biosystems, USA). *GAPDH* and *β-actin* genes were used as endogenous controls to normalise all the threshold cycle (Ct) values. All experiments were performed in triplicate. The relative quantification of gene expression was calculated using the 2^−ΔΔCt^ method and results were expressed as Log 10 (2^−ΔΔCt^) [15]. The data obtained was normalised to the expression of the respective genes in controls and is presented in terms of fold change [15].

### Separation of PBLs from whole blood

PBL were isolated from whole blood of 106 HNSCC patients and 122 controls recruited for this study by density gradient centrifugation using Histopaque 1077 (Sigma-Aldrich) [13]. To 3 mL of Histopaque, equal volume of whole blood was added and centrifuged at 400× g for 30 minutes. The PBL were collected from the interphase and centrifuged twice with phosphate buffered saline. To the PBL pellet, 100 μL of cell lysis buffer and 1× protease inhibitor cocktail (AMRESCO, USA) was added. The isolated PBL were then lysed by freezing and thawing in cell lysis buffer and the total protein content of the PBL lysate was quantified using the method of Bradford.

### Immunoprecipitation of proteins

Prior to Western Blotting, the BRCA2, XPD and APE1 proteins were immunoprecipitated from the PBL lysates of 12 HNSCC and 8 control blood samples as described by Choudhury and Sharan [16], using 1.5 μg of each anti-BRCA2, anti-XPD and anti-APE1 antibodies.

### Western blotting

Western blotting for BRCA2, XPD and APE1 proteins was performed in order to verify the specificity of the antibodies used for the respective proteins, as previously described [13]. For this, immunoprecipitated BRCA2, XPD and APE1 proteins were blotted onto PVDF membrane (Santa Cruz Biotechnology, Inc., USA, 0.45 μm pore size) using Mini PROTEAN Tetra Cell (Bio-Rad, USA).

### Slot blotting

The assay for total BRCA2, XPD and APE1 protein of PBL from 100 HNSCC patients and 122 controls was performed by slot blot immunoprobe assay as described earlier [13]. Slot blotting technique is used for screening a large number of samples as it is quick, efficient and requires less amount of sample [13]. For this, 500 ng of total protein was blotted in triplicate onto PVDF membrane (Santa Cruz Biotechnology, Inc., USA, 0.45 μm pore size) using Bio-Dot SF microfiltration apparatus (Bio-Rad, USA).

### Immunoprobing of western blot and slot blot

Following western blotting and slot-blotting, the membrane was stained with Ponceau S solution (0.1% Ponceau S in 5% acetic acid) to ensure equal loading of protein. The membrane was then washed with distilled water thrice to remove the stain and then incubated with anti-BRCA2 (1:2,000), anti-XPD (1:5,000), anti-APE1 (1:5,000) and anti-β-actin (1:250) antibodies in blocking solution (5% fat free milk in tris-buffered saline (TBS) buffer (10 mM Tris Cl, 500 mM NaCl, pH 7.5)) at 4°C, overnight. Next day the blots were washed in TBS with Tween® 20 (TTBS) solution (0.05% Tween 20 in TBS buffer) for 10 minutes on a shaker at low speed, followed by incubation in alkaline phosphatase labelled goat anti-rabbit IgG (at a ratio of 1:10,000) in blocking solution for 2 hours at 37°C. After incubation, blocking solution was discarded, and the blots were washed in TTBS for 10 minutes, followed by TBS (10 mM Tris-Cl, pH 7.5; 500 mM NaCl) for another 10 minutes. The colour was developed by incubating the membrane in BCIP/NBT colour developer (5–10 minutes) at 37°C.

### Densitometric analysis

The immunoprobed as well as the Ponceau S stained western blots and slot blots were scanned (HP Scanjet G3110) and the images used for densitometric analysis with the help of Image J software (version 1.51e) as described earlier [13]. The staining intensity of the scanned blots was expressed as density of pixels in arbitrary units (AU), such that the greater the pixel density, the more abundant is the protein.

### Classification and regression tree (CART)

CART analysis was performed in order to identify the combination/s of proteins and aetiological factors, viz., BRCA2, XPD, APE1 protein levels, age, gender, smoking and CT habits, which can be used to predict the risk for HNSCC. The CART analysis was performed in R (version 3.3.0) using ‘rpart’ and ‘rpart.plot’ packages. The root node consisting of total sample is split into two sub nodes. The process continues until a full grown tree is generated [17].

### Statistical analyses

Statistical analyses were performed using Statistical Package for the Social Sciences (SPSS) software (version 16.0) and GraphPad Prism (version 5.01) software. Fisher’s exact test or chi square test was performed to determine association between demographic variables, namely, gender, age and aetiological habits. The significance of the difference in level of expression of the *BRCA2, XPD* and *APE1* genes among the peripheral blood, NAT and tumour tissue of HNSCC patients of different stages was determined using one way analysis of variance (ANOVA) with Tukey’s post-hoc test. The significance of the difference in level of BRCA2, XPD and APE1 proteins between HNSCC patients and control was determined using Student’s *t* test as reported earlier [13]. The influence and association of various aetiological factors on the risk for HNSCC was determined using Mann–Whitney rank test. Statistical significance of association between level of BRCA2, XPD and APE1 in blood of HNSCC patients and the different stages of cancer was calculated using Spearman correlation following the methodology reported earlier by Hwang *et al* [18] and Ding *et al* [19]. A *p* value of less than 0.05 was considered to be significant for all the tests.

## Results

### Study subjects

A total of 228 study subjects (106 HNSCC patients and 122 controls) were recruited for this study. Among the patients, those diagnosed with stage III/IV HNSCC at the time of presentation were higher in proportion (33% males and 20.8% females) than those diagnosed with stage I/II and stage 0 of HNSCC. The demographic characteristics of the study subjects are presented in Table 1. The mean and median age of the HNSCC patients (Mean age ± SD = 52.89 ± 14.68, Median age = 52) were found to be higher than that of control individuals (Mean age ± SD = 45.73 ± 16.55, Median age = 45). Incidence of laryngeal cancer (48%) was found to be the highest followed by cancer of the oral cavity (36%) and pharynx (17%), respectively, among the study subjects. The incidences of laryngeal and pharyngeal cancer were higher among males (57.35% and 22.06%, respectively) in comparison to females (31.58% and 5.26%, respectively). However, the incidence of oral cavity cancer was found to be higher among females (63.16%) than among males (20.59%).

### Association of aetiological habits with risk for HNSCC

The study subjects were questioned about the habits of smoking, CT or BN use alone or in combination. Majority (70.27%) of the controls (21.33% males and 48.94% females) reported none of these habits. On the contrary, majority (74.11%) of the HNSCC patients (62.71% males and 11.4% females) reported at least one of these habits. Furthermore, the prevalence of multiple habits of smoking + CT + BN was significantly higher among HNSCC patients (34.90%) than controls (11.48%).

Moderate smokers were at a significantly increased risk for HNSCC (OR = 2.70; 95% confidence interval (CI): 1.33–5.49; *p* < 0.05) in comparison to non-smokers. The risk increased further among heavy smokers (OR = 3.72; 95% CI: 1.93–7.17; *p* < 0.001). Similarly, moderate (OR = 1.91; 95% CI: 1.01–3.61; *p* = 0.053) and heavy CT + BN chewers (OR = 2.15; 95% CI: 1.14–4.04; *p* < 0.05) were at a significantly increased risk for HNSCC. The combined habits of smoking + CT + BN use resulted in the highest risk (OR = 4.14; 95% CI: 2.10–8.16; *p* < 0.001) for HNSCC in the study population.

### Differential mRNA expression of BRCA2, XPD and APE1 genes in tumour tissue, NAT and blood of HNSCC patients

We initially investigated the expression patterns of the *BRCA2, XPD* and *APE1* genes in the matched tumour tissue, NAT and blood samples of 12 out of the 106 HNSCC patients enrolled for this study by quantitative real time-PCR, normalising them against the levels of expression of the genes in the blood samples of 8 age- and gender-matched controls. Two ‘housekeeping’ genes (*GAPDH* and *β-actin*) were used as endogenous controls in the experiment in order to normalise the Ct values of the targeted genes.

**Figure 1.**
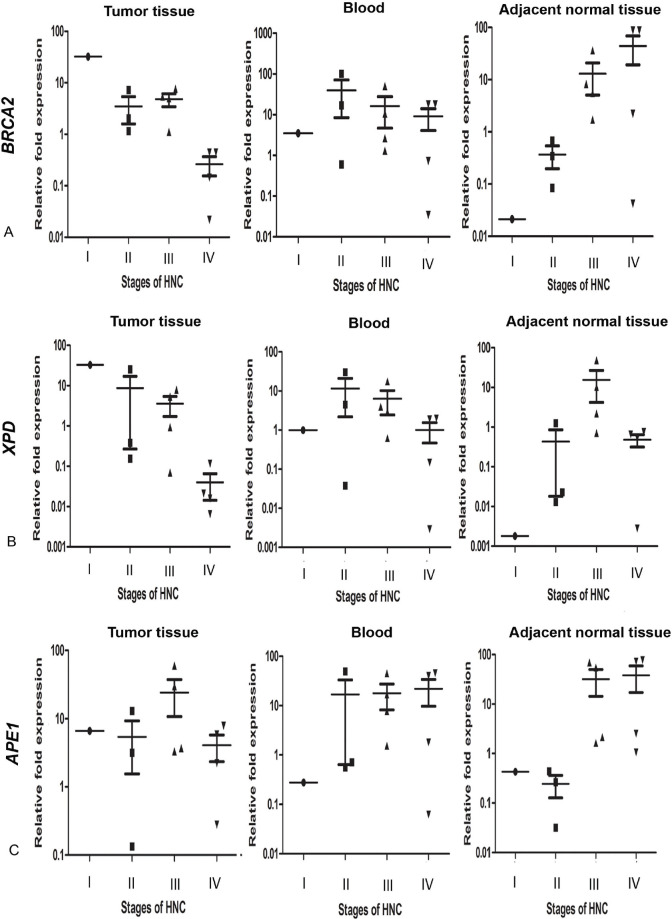
Level of expression of (a): *BRCA2*, (b): *XPD* and (c): *APE1* genes in the tumour tissue, blood and NAT of HNSCC patients, respectively, normalised with level of respective genes in age- and gender-matched control blood samples. AU stands for arbitrary units. Difference of gene expression among the stages was evaluated by one way ANOVA with Tukey’s post-hoc test and was not found to be statistically significant.

Upon stratification of samples on the basis of HNSCC stage, we observed a trend of stage-dependent alteration in the mRNA expression of the three genes. The expression of *BRCA2* and *XPD* in the tumour tissue of HNSCC patients decreased with an increase in the stage of cancer. Interestingly, the trend of specific gene expression observed in NAT was reverse to that of tumour tissue, with the expressions of *BRCA2* and *XPD* genes showing a trend of increasing in the NAT with an increase in cancer stage. While both genes were expressed at a higher level in the blood of HNSCC patients compared to that of corresponding controls, a trend of decline in expression of both BRCA2 and XPD from stage II through stage IV was observed, mirroring the expression pattern observed in the tumour tissue, in both cases. The *APE1* gene did not exhibit a clear trend of expression in tumour tissue with respect to cancer stage. A clear trend of increase in *APE1* gene expression with increase in cancer stage was observed in the blood samples, which resembled that of the NAT rather than the tumour tissue (Figure 1).

Thus, preliminary analysis in a randomly selected subset of HNSCC patients and controls indicated that changes in mRNA expression of the *BRCA2* and *XPD* genes in the tumour tissue of HNSCC patients were mirrored by the matched blood of the patients, such that expression of these two genes exhibited a trend of decrease with an increase in cancer stage. However, these changes were not statistically significant.

### Differential expression of BRCA2, XPD and APE1 proteins in PBLs of HNSCC patients vis-a-vis those of controls

**Figure 2.**
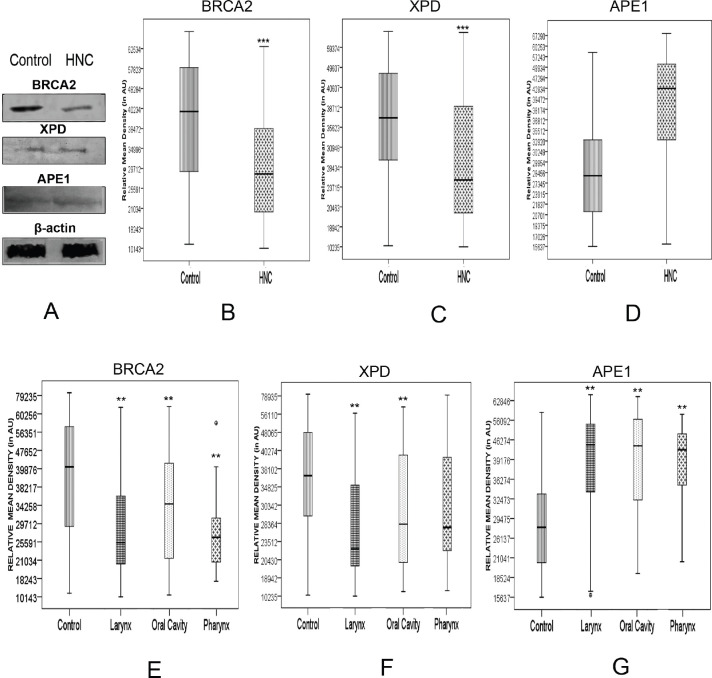
Alterations in the level of BRCA2, XPD and APE1 proteins determined by (a): western blotting technique, with β-actin protein serving as loading control; and slot blotting followed by immunoprobing for (b): BRCA2, (c): XPD and (d): APE1 proteins in the PBLs of HNSCC patients compared to age- and gender-matched controls, with respective Ponceau S stained slot-blots serving as loading control. ** denotes statistical difference between controls and cases at *p* < 0.01; *** denotes statistical difference between controls and cases at *p* < 0.001. AU stands for arbitrary units.

We validated the quantitative reverse transcription PCR (qRT-PCR) results by immunoprobing for BRCA2, XPD and APE1 proteins in the PBL of all 106 HNSCC patients and 122 controls recruited for this study. Western blots immunoprobed for BRCA2, XPD and APE1 proteins confirmed specificity of the antibodies used and thus their suitability for slot blotting (Figure 2). Western blotting also revealed that the levels of BRCA2, XPD and APE1 proteins were differentially regulated in PBL of HNSCC patients with respect to those of controls (Figure 2a).

The slot blotting technique was used to quantify the difference in the expression of BRCA2, XPD and APE1 proteins in the PBL of HNSCC patients in comparison to those of controls. The technique of slot blot is advantageous over western blotting in various aspects. Slot blotting can be used for screening a large number of samples for a particular protein in a very short time. It is thus suitable for samples such as PBL from which proteins can be recovered in small quantities [20]. Moreover, slot blotting can quantify a protein in its native form on the membrane, while it generally gets denatured in SDS gel electrophoresis. This difference is crucial in terms of antigen characteristics of protein [20].

**Supplementary Figure 1.**
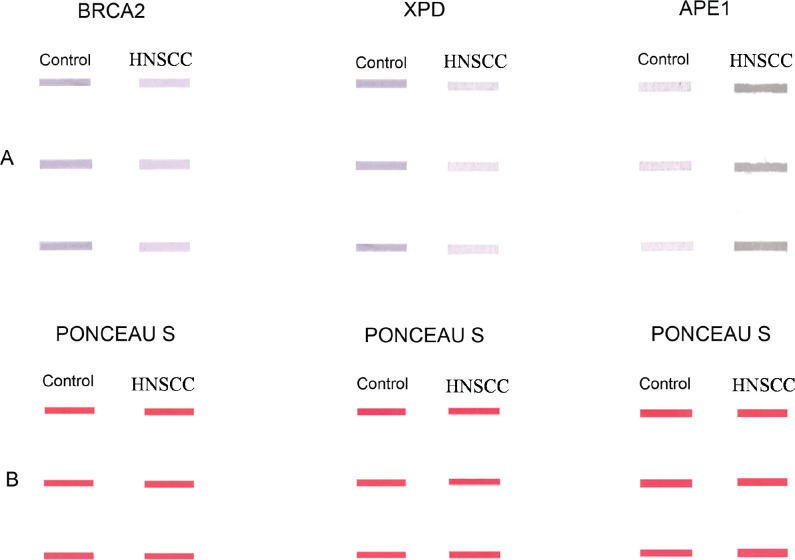
Representative slot-blots showing (a): altered expression of BRCA2, XPD and APE1 proteins in the PBLs of HNSCC patients in comparison to those of age- and gender-matched controls. (b): The corresponding Ponceau S stained slot-blots to verify equal loading of samples.

The expression level of the BRCA2, XPD and APE1 proteins in PBL of HNSCC patients and controls was determined by densitometric analysis of the respective slot-blot [13]. The Ponceau S stained slot blots used as loading control did not show any significant difference in the net pixel density of PBL lysate of HNSCC patients and controls, indicating equal loading of the protein samples. However, the immunoprobed slot-blots revealed significant alteration in expression levels of BRCA2, XPD and APE1 proteins in PBL of HNSCC patients *vis-a-vis* those of controls. BRCA2 and XPD proteins in the PBL of HNSCC patients were significantly (*p* < 0.0001) downregulated to 71% and 77%, respectively, of the level in the PBL of age- and gender-matched controls (Figure 2b and c; Supplementary Figure 1). On the other hand, APE1 protein levels in the PBL of HNSCC patients were significantly (*p* < 0.0001) upregulated to 1.47 fold that in the PBL of age- and gender-matched controls (Figure 2d; Supplementary Figure 1).

**Figure 3.**
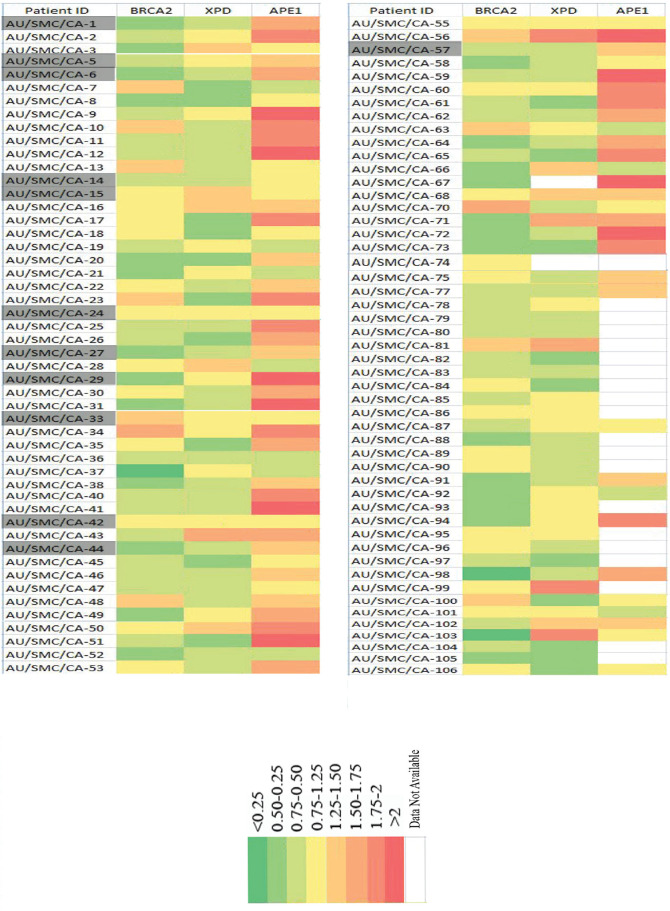
Comparative analysis of the expression profile of BRCA2, XPD and APE1 proteins in the PBLs of individual HNSCC patients normalised with respect to the corresponding levels in the PBLs of age- and gender-matched controls. Each row denotes individual patients containing patient ID and each column represents the fold level of the designated protein. The highlighted patients IDs are the 12 HNSCC patients from whom tissue biopsy, blood and adjacent normal tissue were obtained for real-time analysis study.

A comparative analysis of the expression profile of BRCA2, XPD and APE1 protein levels of individual patients normalised with respect to the levels in age- and gender-matched controls revealed declined levels of BRCA2 and XPD proteins concomitant with elevated level of APE1 in PBL of majority of the patients (Figure 3). We observed that 26.4% of the samples exhibited BRCA2 protein expression levels within a cut off value of 25% (0.75–1.25), which were considered to be within the normal range of expression as per a previous report [13] and a negligible fraction (0.1%) showed slightly upregulated levels (1.25–1.50). However, the remaining 63.10% of the samples showed low levels of BRCA2 expression relative to the levels in the PBL of controls, with 35% exhibiting levels ranging between 0.75 and 0.50 fold that of the control level, 25.24% displaying levels ranging between 0.5 and 0.25 fold that of the control level and 3% exhibiting extremely low levels of BRCA2 protein (<0.25 fold of controls).

Similarly, 27.72% of the samples analysed exhibited XPD protein levels within the normal range of expression (0.75–1.25) with respect to levels in the PBL of corresponding age- and gender-matched controls. Close to 10% of the samples exhibited higher levels of XPD than those observed in the PBL of corresponding controls. Among the remaining samples, 42.57% exhibited XPD protein levels within a range of 0.75–0.50 fold that of control level, and 19.8% samples exhibited XPD protein levels within a range of 0.50–0.25 fold that of corresponding controls.

Contrary to the expression pattern of BRCA2 and XPD proteins, we observed a trend of overexpression of the APE1 protein, with 17.4% samples exhibiting 1.25–1.50 fold, 12.79% samples exhibiting 1.50–1.75 fold, 16.28% samples exhibiting 1.75–2 fold and 13.95% samples exhibiting more than two fold increase in the expression of APE1 protein in the PBL, compared to the corresponding levels of the protein in the PBL of age- and gender-matched controls. Among the remaining samples, 22.09% expressed APE1 within the normal cut-off range (0.75–1.25), and 13.95% exhibited low (0.75–0.50) levels of APE1 protein.

Among the 14 patients recruited for the gene expression study by real-time PCR, all 4 patients (28.57%) of stage IV HNSCC (AU/SMC/CA-1, AU/SMC/CA-6, AU/SMC/CA-27 and AU/SMS/CA-44; Figure 3) exhibited low levels of expression of the *BRCA2* gene in matched tumour and blood samples, concomitant with low levels of the BRCA2 protein in the PBL.

### Correlation between cellular levels of BRCA2, XPD and APE1 proteins in PBL of HNSCC patients and different stages of HNSCC

Spearman correlation analysis was performed in order to determine the association between the level of expression of the BRCA2, XPD and APE1 proteins in the blood sample of HNSCC patients with the different stages (I, II, III and IV) of HNSCC. A significant (*p* < 0.01) negative correlation was obtained for BRCA2 (*p* < 0.0001) and XPD (*p* < 0.01) with correlation coefficient (*r*_s_) of −0.9060 and −0.8008, respectively. However, for APE1, a significant (*p* < 0.05) positive correlation coefficient (*r*_s_) of 0.7023 was observed (Table 2). Thus, the levels of BRCA2 and XPD in PBL of HNSCC patients were found to be downregulated, while the levels of APE1 were upregulated with an increase in the stage of HNSCC, in comparison to the levels in the PBL of age- and gender-matched controls (Table 2).

### CART analysis

The CART analysis was used to illustrate associations between variables which are not suited to traditional regression analysis. CART overcomes missing data by use of surrogate measures and helps to identify previously unknown pattern among data [21]. Moreover, in multimarker studies, logistic regression helps to determine the relative statistical significance of the markers employed, whereas CART is more focused on the actual difference between groups and helps to identify risk [22]. Thus, A CART model was generated to determine the association between different factors investigated and the risk for HNSCC.

**Figure 4.**
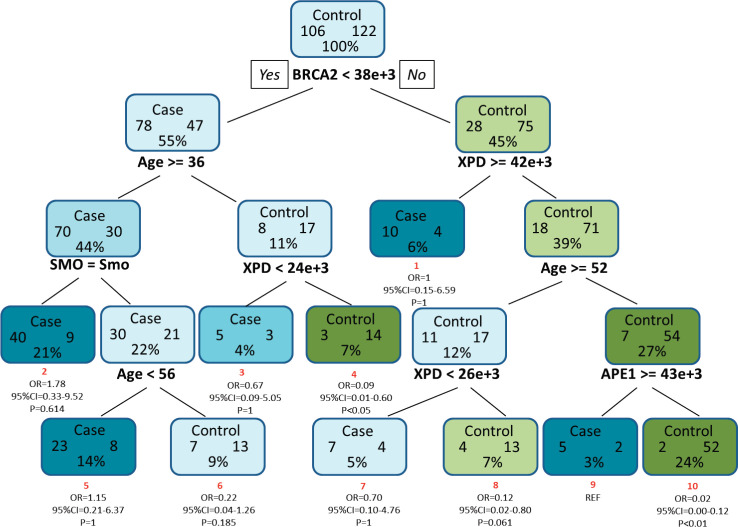
CART analysis for determining the association between different factors in increasing the risk for HNSCC. The tree consists of ten terminal nodes (numbered in red) along with their respective OR, 95% CI and p value. Each node consists of the total number of cases and controls along with the percentage of total sample. SMO and Smo denote smoking status and smokers, respectively.

For analysis of protein–protein and protein–environment interactions, a final tree consisting of ten terminal nodes was generated. The first split of the root node was based on the level of BRCA2 clearly indicating that levels of BRCA2 in the PBL can serve as an indicator of HNSCC risk. Low levels of BRCA2 protein were indicated as is the single most important risk factor for HNSCC. Among study subjects with mean intensity of BRCA2 less than 38,000, the tree was further split to show interaction among age, smoking and level of XPD protein. Individuals with mean intensity of BRCA2 above 38,000, showed an interaction between level of XPD, age and level of APE1. Terminal node 9 was considered as the reference node for calculating the odds ratio (OR) as it contained the least percentage of samples. Subjects with low levels of BRCA2 in the PBL (<38,000), greater than 36 years of age and the habit of smoking (terminal node 2), exhibited a 1.78-fold risk for HNSCC (OR = 1.78, 95% CI = 0.33–9.52), though this risk was not significant statistically. Risk was also observed for terminal node 5 where non-smokers aged between 36 and 56 years, with low levels of BRCA2 had a moderate (OR = 1.15, 95% CI = 0.21–6.37) but non-significant risk for HNSCC (Figure 4).

## Discussion

The BRCA2 protein is an essential part of the HR repair system and maintains genomic integrity through its physical and functional interaction with RAD51, and in turn, p53 via the p53 mediated pathway [23]. Reduced expression of BRCA2 has been reported to play a vital role in promoting tumorigenesis [24], but its role in head and neck carcinogenesis has not been defined. We observed a trend for stage-dependent decline in *BRCA2* gene expression in the tumour tissues of HNSCC patients, while the matched NAT showed the reverse trend of significantly increasing with the progression of cancer stage. The expression of BRCA2 protein in the PBL of HNSCC patients also declined significantly to 71% of the level in PBL of controls and exhibited a significant negative correlation with cancer stage. Fanale *et al* [25] previously reported a downregulation of BRCA2 protein in breast cancer cell line. In previous *in vivo* studies using animal models, we have reported that decline in tissue BRCA2 levels is an important mechanism of smokeless tobacco [26] and BN [16] induced carcinogenesis and was mirrored by PBL of carcinogen exposed animals in the latter [16]. In another study, the DNA damaging UV irradiation, γ-irradiation, adriamycin and camptothecin were shown to significantly enhance the rate of degradation of BRCA2 mRNA and to reduce the stability of BRCA2 protein [27].

The helicase XPD is part of the structure of transcription factor IIH (TFIIH), and its adenosine triphosphate (ATP)-dependent helicase activity along with the ATPase activity of another protein, XPB, is essential for anchoring TFIIH to the site of DNA damage followed by opening of the DNA duplex flanking the lesion, during NER [28]. In a previous study, XPD transfected into the hepatocellular carcinoma cell line, HepG2, displayed tumour suppressive effects [29]. In this study, XPD mRNA and protein exhibited a trend of expression similar to BRCA2. The level of *XPD* mRNA expression in the tumour tissue of HNSCC patients decreased with increasing stage of cancer, as did the expression of XPD protein in the PBL of HNSCC patients which showed an overall significant decline to 77% of the level in PBL of controls. Our findings are consistent with those of Lin *et al* [30], who reported a downregulation of *XPD* gene expression in HNSCC tissues. Other studies have also reported the reduced expression of the *XPD* gene in HNSCC [31] and an increased risk for HNSCC with the reduced expression of XPD [32].

APE1 is a multifunctional protein which not only plays a central role in BER, but is a central hub in protein–protein interactions connecting various subnetworks involved in tumour progression, chemoresistance, RNA- and DNA-metabolism. The overexpression of APE1 is associated with chemoresistance, and BRCA emerged as an important interacting partner with APE1 in a systems biology approach used to study the APE1 interactome [33]. Previous studies have reported increased expression of APE1 in bone marrow stromal cells of multiple myeloma [34], head and neck cancer, rhabdomyosarcomas, bladder, ovarian, gastro-esophageal and pancreatico-biliary cancers, prostate, thyroid and hepatocellular cancers and upregulated expression levels associated with poor prognosis in medulloblastoma [35]. In this study, we observed a significant 1.47-fold increase in the level of APE1 protein in the PBL of HNSCC patients in comparison to controls. However, we did not observe a clear trend in the level of *APE1* gene expression in the tumour tissue of HNSCC patients. The overexpression of APE1 would enhance the activity of various transcriptional factors, leading to promotion of growth, migration and survival in tumour cells and increased aggressiveness [33, 36].

An interesting finding of our study is the trend of *BRCA2* and *XPD* expression in the NAT of HNSCC patients, which was found to be the reverse of those in the tumour biopsies and blood of the same patients (Figure 1). A tumour cell affects its microenvironment, thereby also modulating its ability to grow. The ability of the normal cell to respond to the various factors secreted by the tumour cells plays a crucial role in the manifestation of cancer [37]. In fact, a previous study has reported that the NAT presents an intermediate state between healthy and tumour tissue and several pathways are altered in NATs across different tissue types [38]. In our study, the expressions of *BRCA2* and *XPD* are probably elevated in tumour tissue and blood of patients in the early stages of cancer in an effort to increase the DNA repair capacity, which is essential for promoting tumour growth, and the cancer phenotype [39]. The progressive decline in both BRCA2 and XPD mRNA and protein expression may be a result of carcinogen exposure due to rampant tobacco use by the HNSCC patient group, but are compensated by an increase in APE1 expression. However, an opposite effect was observed in the adjacent normal cells. Hence, it is hypothesised that while the adjacent normal cells in the stage II and stage III HNSCC patients had elevated *APE1* expression, they also upregulated *BRCA2* and *XPD* expression under the influence of the tumour cells in an effort to suppress their own carcinogenic transformation. This hypothesis may be the subject of further study, but a compensatory effect similar to the one observed by us was reported in HPV-positive cell lines which were found to have reduced levels of DSB repair proteins including BRCA2, but conversely upregulated levels of proteins involved in BER and single strand break repair [40].

The HPV-positive oropharyngeal squamous cell carcinoma cell line, UPCI-SCC90 [40], and the HNSCC cell line, UPCI-SCC154 [41], were reported to harbour reduced levels of BRCA2 and other DNA repair proteins, in comparison to HPV-negative cell lines [40, 41]. This deficiency in DNA repair activity resulted in higher radiosensitivity of UPCI-SCC90 [40] and increased sensitivity of UPCI-SCC154 to the PARP inhibitor, veliparib [41]. Previous studies have found a significant association of HPV infection with oral squamous cell carcinoma in Northeast India [42–44], concomitant with a strong correlation with p16 overexpression [42]. While we have not ascertained the HPV or p16 status of our study samples, it is probable that the reduced BRCA2 levels observed in our study may also be associated with HPV-positivity of the samples, though this conjecture has to be further investigated.

Although gene expression results have found their usefulness in the many applications like diagnosis and classification of cancers, the results are undoubtedly more correlative rather than causative. It is most likely the concentration of the proteins and their interactions with each other that are the true causative forces in the cell [45]. Moreover, due to the invasiveness of tissue biopsies, the difficulties posed for repeated sampling and the unavailability of large number of tissue biopsies, we have emphasised on evaluating the expressions of BRCA2, XPD and APE1 in the PBL of HNSCC patients and control individuals, using slot blot technique for validating our result on a larger sample size. PBL being the first responder to foreign attack also participates in cellular and extracellular matrix communication among different organs and tissue of the body [46] and is likely to exhibit patterns of gene and protein expression which reflect phenotypic changes such as cancer development and/or progression in tissues. CART analysis revealed an increased risk for HNSCC among smokers with low XPD and BRCA2 levels, underlining the significance of BRCA2 and its interaction with tobacco-derived carcinogens in the pathogenesis of HNSCC.

DNA repair processes play an important role in determining the response to therapy, and BRCA2 is an important regulator of the response to commonly used platinum-based anticancer drug, cisplatin. Antisense oligonucleotide mediated knockdown of BRCA2 increased the sensitivity of human lung, ovarian and breast cancer cells to cisplatin and reversed the resistance to cisplatin acquired by head and neck cancer cells. Furthermore, knockdown of BRCA2 after cisplatin treatment also decreased the metastasis of tumours, *in vivo* [47]. Thus, decreased levels of BRCA2 protein observed in our study could also have therapeutic implications in terms of patient response to platinum-based therapy and may be studied further.

## Conclusions

In conclusion, our study indicates that the pathogenesis of HNSCC involves a decline, albeit non-significant, of BRCA2 and XPD-mediated DNA repair functions along with dysfunctional APE1 response. We also propose that levels of BRCA2 in PBLs may be useful for monitoring the risk for HNSCC, with low levels of BRCA2 among smokers indicating a high risk for HNSCC. A limitation of this study is that we did not account for interpersonal variation in leucocyte composition of the blood samples collected and did not ascertain the HPV status or p16 expression of the study samples. Furthermore, the associations observed exhibited clear trends, but were not statistically significant probably due to the sample size of the study.

## List of abbreviations

APE1, Apurinic/apyrimidinic endodeoxyribonuclease 1; BER, Base excision repair; BN, Betel-nut; BQ, Betel quid; BRCA2, Breast cancer susceptibility 2; CART, Classification and regression tree; CI, Confidence interval; CT, Chewing tobacco; HNSCC, Head and neck squamous cell carcinoma carcinoma; HPV, Human papillomavirus; HR, Homologous recombination; NAT, Normal adjacent tissue; NER, Nucleotide excision repair; NNK, 4-methylnitrosoamino-1-(3-pyridyl)-1-butapone; NNN, N-nitrosonornicotine; OR, Odds ratio; PBL, Peripheral blood lymphocytes; XPD, Xeroderma pigmentosum group D.

## Conflicts of interest

The authors declare that they have no conflicts of interest.

## Funding

This work was funded by grant with sanction no. BT/CP/09/NE/TBP/2010 dated 20 October 2011, from the Department of Biotechnology, Government of India to Yashmin Choudhury and Sankar Kumar Ghosh (grant period 2011–2015). Sambuddha Das, Aditi Bhowmik and Bishal Dhar were supported by University Grants Commission-Basic Scientific Research fellowship. The authors’ would also like to acknowledge the Department of Biotechnology, Government of India for providing Real-Time PCR facility ((BT/01/NE/TBP/2011(Med)) in the Molecular Medicine Laboratory, Department of Biotechnology, Assam University.

## Figures and Tables

**Supplementary Table 1:** Table showing sequence of primers for Real Time PCR

Gene	Primer sequence
*BRCA2*	Forward 5'-CTTCATAAGTCAGTCTCATCTG-3'
	Reverse 5'-GTCTTGCGTTTTGTAATGAAG-3'
*XPD*	Forward 5'-CAAAGAGACAGACGAGCAGCGC-3'
	Reverse 5'-TTCGTCGGGCAGCACGGGG-3'
*APE1*	Forward 5'-GCTGCCTGGACTCTCTCATC-3'
	Reverse 5'-AAACTTTGAGTGGGCACTGG-3'
*GAPDH*	Forward 5'-GAAATCCCATCACCATCTT-3'
	Reverse 5'-GAGCCCCAGCCTTCTCCA-3'
*Β-ACTIN*	Forward 5'-TGGATCAGCAAGCAGGAGTATG-3'
	Reverse 5'-AAGAAAGGGTGTAACGCAACTAA-3'

**Table 1.** Demographic characteristics of controls and HNSCC patients recruited for the study.

Characteristic		Controls (%)		Cases (%)	*p* value
Sex					
Male		75 (61.47)		68 (64.15)	1^a^
Female		47 (38.53)		38 (35.85)	
Age					
<30		25 (20.49)		4 (3.77)	<0.001^b^
30–60		72 (59.02)		70 (66.04)	
>60		25 (20.49)		32 (30.19)	
Mean age ± SD		45.73 ± 16.55		52.89 ± 14.68	
Median age		45		52	
Aetiological habits					
Smoking	Male		16 (21.33)	22 (32.26)	<0.001^b^
	Female		6 (12.77)	2 (5.26)	
CT + BN	Male		31 (41.33)	10 (14.52)	
	Female		16 (34)	23 (60.53)	
Smoking + CT + BN	Male		12 (16.01)	33 (48.38)	
	Female		2 (4.29)	5 (13.16)	
None	Male		16 (21.33)	3 (4.84)	
	Female		23 (48.94)	8 (21.05)	
Site of occurrence of HNSCC					
Oral cavity	Male	NA		14 (20.59)	
	Female			24 (63.16)	
Pharynx	Male	NA		15 (22.06)	
	Female			2 (5.26)	
Larynx	Male	NA		39 (57.35)	
	Female			12 (31.58)	
Stage of HNSCC					
Stage 0 (premalignant)	Male	NA		7 (6.6)	
	Female			3 (2.8)	
Stage I/II	Male	NA		26 (24.5)	
	Female			13 (12.3)	
Stage III/IV	Male	NA		35 (33)	
	Female			22 (20.8)	

NA, Not applicable

a Fisher’s exact test

b Chi-square test

**Table 2.** Correlation between levels of BRCA2, XPD and APE1 proteins and different stages (I, II, III, IV) of HNSCC.

Proteins	Correlation coefficient (*r*_s_)^a^	95% confidence interval	*p* value
BRCA2	−0.9060	−0.9746 to −0.6814	<0.0001
XPD	−0.8008	−0.9440 to −0.4036	<0.01
APE1	0.7023	0.1964 to 0.9129	<0.05

a Spearman correlation coefficient
